# Expanding the Analytical Toolbox for the Nondenaturing Analysis of siRNAs with Salt-Mediated Ion-Pair Reversed-Phase Liquid Chromatography

**DOI:** 10.1021/acs.analchem.4c05248

**Published:** 2024-11-11

**Authors:** Martin Enmark, Ilaria Furlan, Porya Habibollahi, Christian Manz, Torgny Fornstedt, Jörgen Samuelsson, Eivor Örnskov, Manasses Jora

**Affiliations:** † Department of Engineering and Chemical Sciences, Karlstad University, Karlstad 651 88, Sweden; ‡ Advanced Drug Delivery, Pharmaceutical Sciences, BioPharmaceuticals R&D, AstraZeneca, Mölndal 431 83, Sweden; § Medicinal Chemistry, Research and Early Development, Respiratory and Immunology, BioPharmaceuticals R&D, AstraZeneca, Mölndal 431 83, Sweden

## Abstract

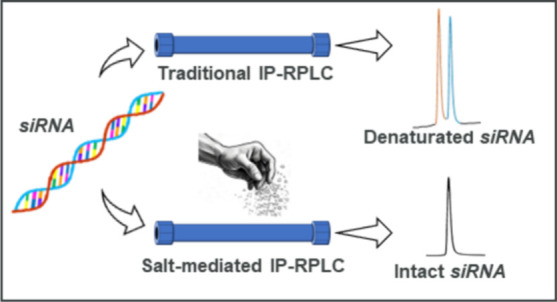

Short interfering RNA (siRNA) represents a rapidly expanding class of marketed oligonucleotide therapeutics. Due to its double-stranded nature, the characterization of siRNA is twofold: (i) at the single-strand (denaturing) level for impurity profiling and (ii) at the intact (nondenaturing) level to confirm duplex formation and quantify excess single strands (including single strand-derived impurities). While denaturing analysis can be carried out using conventional ion-pair reversed-phase liquid chromatography (IP-RPLC), nondenaturing characterization of siRNA is a significantly less straightforward task. Typical IP-RPLC conditions have an intrinsic denaturing effect on siRNA, thereby limiting the development of viable approaches for the intact duplex analysis. In this study, we demonstrate, through the design of experiments of siRNA melting temperatures and chromatography analyses, that the simple addition of salts, such as phosphate-buffered saline and ammonium acetate, to eluents enhances the suitability of IP-RPLC for the nondenaturing analysis of siRNA during both UV- and mass spectrometry-based analysis. This work represents a milestone in overcoming the challenges associated with nondenaturing analysis of siRNAs by IP-RPLC and offers a fresh angle for exploring IP-RPLC of siRNAs.

Short interfering ribonucleic acid (siRNA) is a synthetic double-stranded oligonucleotide (ON) composed of complementary sense and antisense strands, each approximately 18–25 nucleotides long. Due to its ability to regulate gene expression, siRNA has successfully reached the therapeutic market.^1^ Currently, 6 siRNAs (*i.e*., Patisiran, Givosiran, Lumasiran, Inclisiran, Nedosiran, and Vutrisiran) have been approved by the US FDA.

Analytical characterization of ONs is challenging due to the relatively large number of synthesis- and degradation-derived impurities.^2^ Quality control of siRNA is a particularly demanding task as two complementary approaches are required for siRNA characterization:^3^ (i) a denaturing method, in which the duplex is dissociated and impurities are assessed at the single strand level, and (ii) a nondenaturing method, used to confirm duplex formation and quantify excess single strands, including single strand-derived impurities.

Ion-pair reversed-phase liquid chromatography (IP-RPLC) is the method of choice for denaturing analysis.^2^ The wide range of ion-pairing reagents (IPRs), stationary phases, etc. available for IP-RPLC allows it to be tuned to separate even closely related ONs.^2,4^ In addition, it can be coupled to mass spectrometry (MS), allowing direct characterization of ONs.^5^ On the other hand, the application of IP-RPLC as a nondenaturing method is suboptimal.^6^ IP-RPLC has an inherent tendency to denature duplexes even at column temperatures significantly below their melting temperatures (*T*_m_).^7^ Therefore, methods based on hydrophilic interaction chromatography (HILIC),^8^ size exclusion chromatography (SEC),^7,9^ and anion exchange chromatography (AEX)^10^ are usually required to address the limited scope of IP-RPLC as a nondenaturing approach.

Here we evaluate if the addition of salt (*i.e.*, phosphate-buffered saline, PBS, or ammonium acetate, AA) to IP-RPLC mobile phases can help change this paradigm and expand the IP-RPLC toolbox in the context of intact siRNA characterization. Triethylamine (TEtA) and tributylamine (TBuA) were selected as model IPRs. TEtA is traditionally employed in nondenaturing analysis of siRNAs by IP-RPLC, while the latter is not, due to its inherent denaturing properties.

As illustrated in Figure 1a, IP-RPLC diluents can drastically impact the *T*_m_ of siRNA#1. *T*_m_ decreases from 75.4 °C in 1X PBS aqueous solution (green line, a representative condition used to measure *T*_m_ of siRNAs^11^) to 24.2 °C in 16.8 mM tributylaminium acetate (TBuAA), 16.8 vol % acetonitrile (MeCN, blue line, a representative IP-RPLC diluent). This is known^7,12^ and forms the basis for the challenging aspects of nondenaturing analysis of siRNA by IP-RPLC. The higher the impact of IP-RPLC diluents on *T*_m_, the lower the column temperature required for nondenaturing analysis.^7^

**Figure 1:**
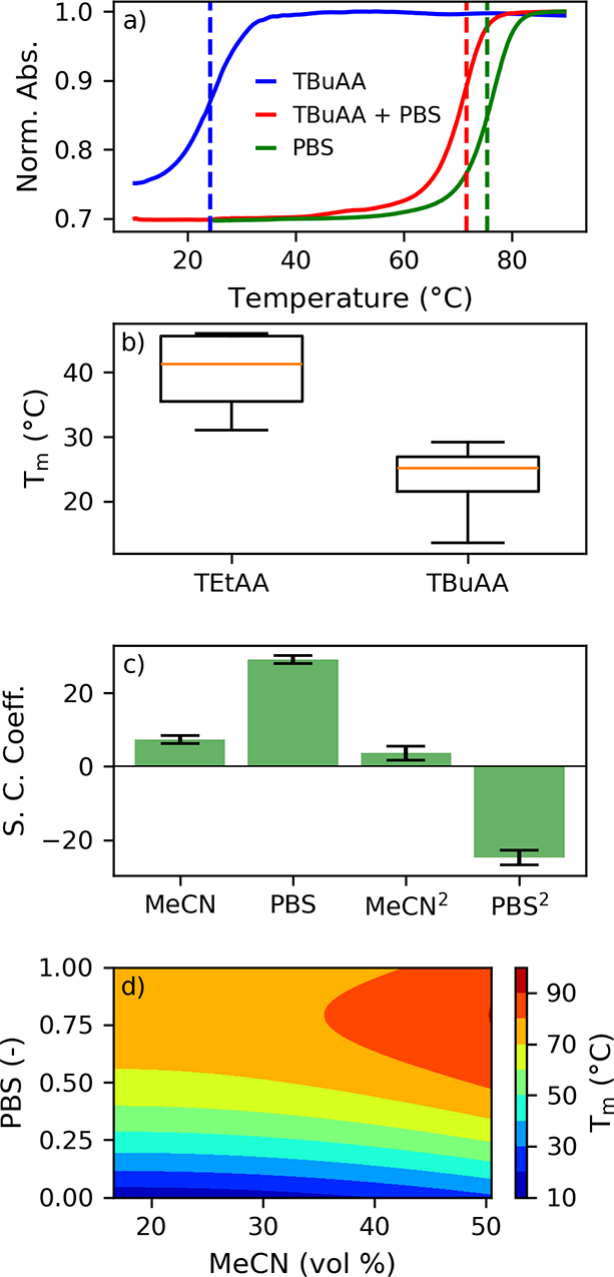
(a) Melting curves of siRNA#1 in 1X PBS (green line), in a representative IP-RPLC diluent (16.8 mM TBuAA, 16.8 vol % MeCN, blue line), and in the same IP-RPLC diluent with the addition of 1X PBS (red line). (b) Box plot representation of the impact of conventional TEtAA- and TBuAA-based diluents on the *T*_m_ of siRNA#1 (Table S1). (c) Scaled and centered coefficients for the DoE model. (d) *T*_m_ contour plot for the DoE model.

It is common knowledge that the *T*_m_ of siRNAs is affected in distinct ways by different IPRs.^7^ As shown in Figure 1b (Table S1), TBuAA-based diluents (*i.e.*, 4.2 and 16.8 mM TBuAA in 16.8 and 50.5 vol % MeCN) cause a more significant decrease on the *T*_m_ of siRNA#1 than triethylaminium acetate (TEtAA)-based diluents (*i.e.*, 10.6 and 83.0 mM TEtAA in 16.8 and 50.5 vol % MeCN) (Figure 1b, Table S1). *T*_m_ values under the former are, in general, lower than those under the latter. This underscores the preference for TEtA, as well as hexylamine (HA),^7^ as the IPRs of choice for nondenaturing IP-RPLC analysis of siRNAs.^9,12^

Preliminary experiments revealed that the addition of PBS into the diluents significantly mitigated the impact of IP-RPLC conditions on the *T*_m_ of siRNA#1. Under similar TBuAA-based diluents, the decrease in *T*_m_ went from 51.2 °C (Figure 1a, green and blue traces) to 3.8 °C (Figure 1a, green and red traces) upon the addition of 1X PBS. To better understand this positive impact of PBS, we systematically measured the *T*_m_ of siRNA#1 under representative IP-RPLC diluents, with and without PBS (Table S2). Outcomes were then modeled by using a two-level full-factorial DoE. TBuAA was used as the model system because of its more significant impact on the *T*_m_ of siRNA#1 (Figure 1b).

PBS and MeCN had a nonlinear effect on *T*_m_ values in relation to their respective concentration in the diluents. Therefore, their quadratic terms were required. To model these quadratic terms, additional experimental data were included in the DoE (Table S2). These resulted in a model fit with coefficient of determination (*R*^2^) of 0.998 and predicting power (*Q*^2^) of 0.994. Scaled and centered coefficients for the DoE model are presented in Figure 1c. They indicate that only the terms involving PBS level and vol % MeCN are significant to the model. Figure 1d presents a contour plot describing how *T*_m_ varies depending on PBS level and vol % MeCN. It is important to note here that despite minor ( ≤ 10%), diluent evaporation might have overestimated the significance of MeCN to the model. In fact, evaporation may have hindered the true impact of MeCN on *T*_m_ of siRNA#1. As recently shown by Gilar *et al.* using differential scanning calorimetry, the addition of cosolvents slightly reduces the *T*_m_ of an siRNA.^7^ Despite the minor difference in outcomes, our results reinforced that PBS substantially mitigates the impact of IPRs on the *T*_m_ of siRNA#1.

Motivated by this positive effect of PBS on the *T*_m_ of siRNA#1 under static conditions, we performed chromatographic experiments to further evaluate if this would hold true in a more dynamic environment, *i.e*., during chromatographic separation. Two sets of 10.6 mM TBuAA IP-RPLC experiments were conducted: one without PBS in the mobile phases and the other with PBS added. Gradient 1 was implemented for experiments without PBS. According to the DoE model, the *T*_m_ of siRNA#1 is ∼25 °C at conditions representing the beginning of the gradient (*i.e*., 47 vol % MeCN). Running the separation at 8 °C (*i.e*., significantly below the predicted *T*_m_) returned a chromatogram with most of the duplex denatured into its corresponding sense and antisense strands (Figure 2a). Features of Figure 2a indicate that the experimental *T*_m_ of siRNA#1 is actually lower than the predicted ∼25 °C. This is likely because of (a) differences between nominal and experimental column temperatures (as mobile phases were neither precooled nor kept in a refrigerated environment) and (b) MeCN evaporation during melting curve experiments (which biased *T*_m_ toward higher values). At higher temperatures (*e.g*., 50 °C, Figure 2a), only single strands were observed. These further illustrate that, at preferential chromatographic temperatures, TBuAA-based IP-RPLC (an example of alkylamine other than the traditionally used TEtA and HA pair) is not suitable for the nondenaturing analysis of siRNA duplexes.

**Figure 2:**
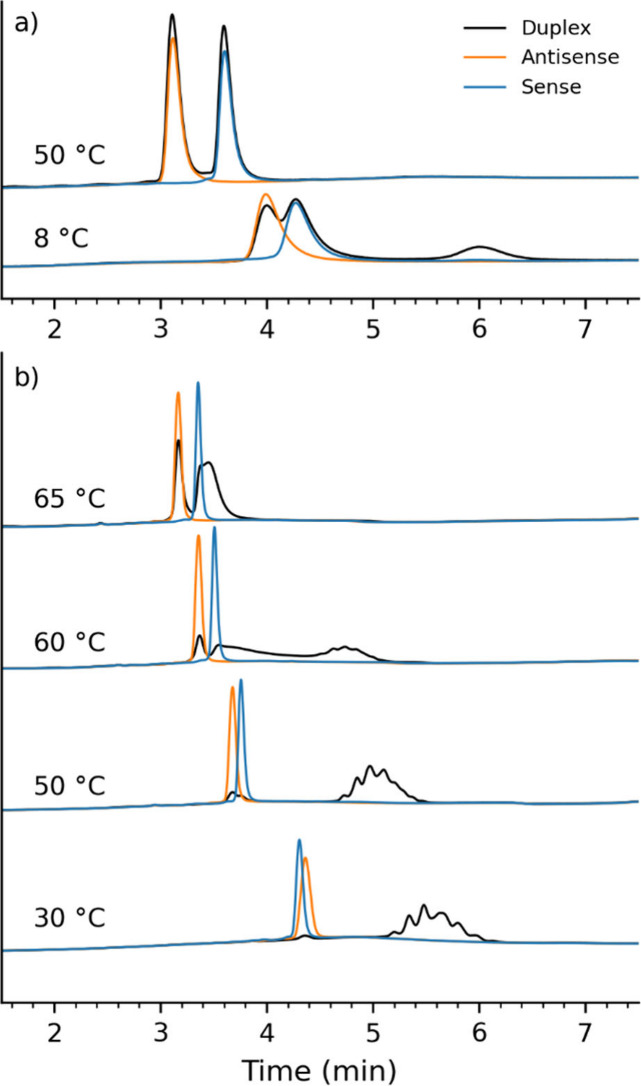
Elution profiles at 260 nm of individually analyzed siRNA#1 duplex (black), sense (blue), and antisense (orange) samples. (a) Profiles were acquired without PBS added to the mobile phases at column temperatures of 8 and 50 °C. (b) The equivalent injections were performed using mobile phases containing 0.25X PBS at column temperatures of 30, 50, 60, and 65 °C.

Preliminary tests revealed that the addition of PBS to the IP-RPLC mobile phases decreased the retention of ONs (data not shown). The reason for this is that IP-RPLC can be thought of as a dynamic ion exchanger and that the presence of salt in the eluent reduces the interaction between the IPR and the analyte.^13^ Therefore, for experiments with PBS, gradient 2 was implemented. According to the DoE model, and for 0.25X PBS, *T*_m_ of siRNA#1 ranges between 47 and 53 °C throughout the gradient. Indeed, the siRNA#1 duplex was kept intact by running the analysis at a temperature below the 47–53 °C range (*e.g*., 30 °C, Figure 2b). Moreover, the expected excess of antisense could be confirmed at this condition. Nonetheless, there were slightly different levels: 1.2% expected (by traditional TEtA-based IP-RPLC; data not shown) versus 2.4% by salt-mediated IP-RPLC (Figure 2b, 30 °C). While not further investigated here, this difference might have been derived from the use of different instruments and software tools for data acquisition and processing of these two data sets, or even due to a potential difference in molar extinction coefficient of the ON species under these different chromatographic conditions. It was also noticed that while the antisense and sense elution peaks were symmetrical and narrow, the chromatographic peak corresponding to the duplex was broad and partially resolved into multiple peaks. This indicates that, in addition to enhancing duplex stability, PBS also promotes diastereomer separation.^2^ By running the analysis at temperatures either within or above the predicted 47–53 °C range, minor/major siRNA duplex denaturation was observed (Figure 2b). At 50 °C, chromatographic peaks corresponding to both sense and antisense strands were detected, indicating the partial melting of siRNA#1. At 60 °C, a broad peak between those of the single strands and the duplex was observed, indicating a more substantial denaturation of siRNA#1. At 65 °C, siRNA#1 was almost completely denatured. These results confirmed that PBS continues to mitigate duplex denaturation even in the dynamic environment of the chromatographic separation, provided that the chromatographic separation is performed significantly below the duplex’s *T*_m_ under the IP-RPLC diluent used for the analysis.

These outcomes were further challenged by repeating the same experiments on a recently approved siRNA (*i.e.*, Inclisiran). The *T*_m_ of Inclisiran is higher than that of siRNA#1 (87.4 °C vs 75.4 °C in 1X PBS); therefore, it is a good model to further test the approach. Like siRNA#1, Inclisiran did not denature at 30 °C (Figure S2). In fact, and unsurprising due to its higher *T*_m_, Inclisiran remained intact also at 50 °C. Therefore, this confirms that the nondenaturing benefits of salt-mediated IP-RPLC observed for siRNA#1 are also applicable to other siRNAs.

Despite being beneficial to the nondenaturing characterization of siRNAs, PBS is not compatible with MS analysis. Salts within PBS have an elevated boiling point. Therefore, it was of interest to test if a MS friendly salt (*i.e.*, AA) would maintain the same nondenaturing conditions as observed for PBS. More importantly, we were interested in testing if IP-RPLC using AA instead of PBS would make the detection of intact duplexes by MS possible.

To this end, siRNA#1 was analyzed by liquid chromatography-mass spectrometry (LC-MS) using the nondenaturing conditions described earlier (*i.e*., gradient 2, 30 °C). The only difference was that 39.8 mM AA was added to the mobile phases, which is equivalent, in terms of ionic strength, to the 0.25X PBS used earlier. Except for differences in elution time, the results using AA were similar to those shown for PBS (Figures 3a and S2). Therefore, this confirmed that AA can also be used for the IP-RPLC-based nondenaturing analysis of siRNA.

**Figure 3:**
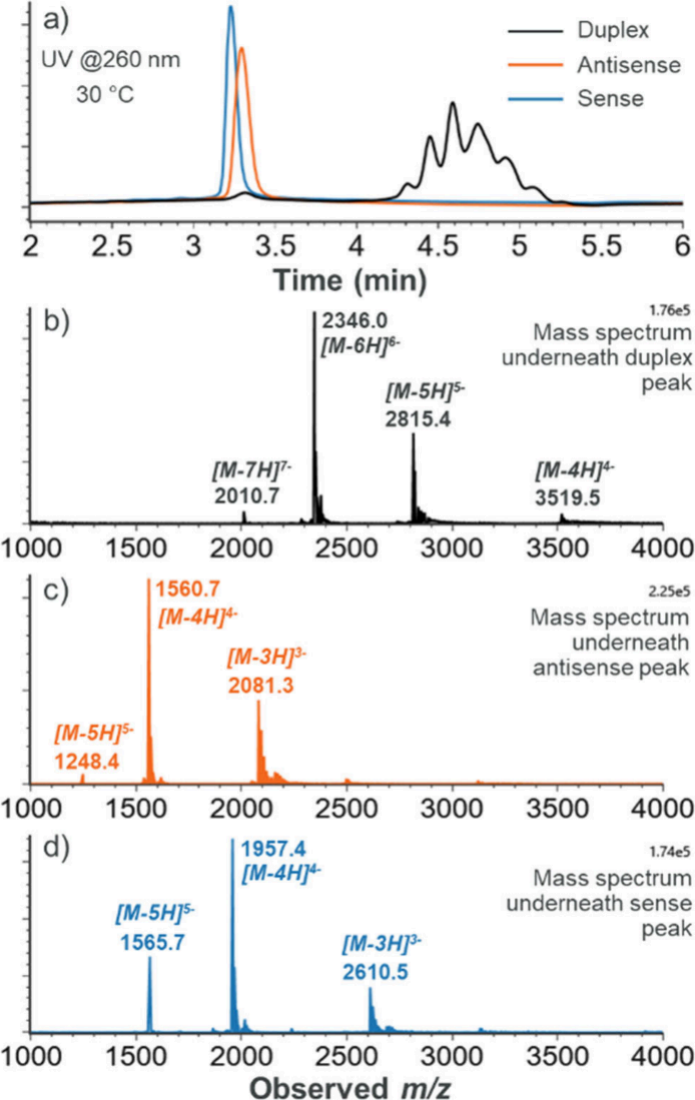
(a) Elution profiles at 260 nm of individually analyzed siRNA#1 duplex (black), antisense strand (orange), and sense strand (blue) samples obtained with 39.8 mM AA in the mobile phases. Mass spectra observed underneath the chromatographic peaks corresponding to (b) siRNA#1 duplex, (c) siRNA#1 antisense strand, and (d) siRNA#1 sense strand. The intensity of each mass spectrum was normalized according to the most abundant peak within each individual mass spectrum and within the 1000–4000 mass-to-charge (*m*/*z*) range.

Furthermore, results confirmed that siRNA#1 is also kept intact during MS analysis. As illustrated in Figure 3b (and Figure S3), no ions corresponding to the single strands were detected underneath the chromatographic peak corresponding to the siRNA#1 duplex. Moreover, siRNA#1 could be directly characterized by the approach. The deconvolution of the mass spectrum for the siRNA#1 duplex yielded an experimental average mass of 14082.74 Da (Figure S4), representing a mere 1.4 ppm mass error in comparison to the theoretical mass of siRNA#1. These LC-MS results suggest that salt-mediated IP-RPLC has the potential to allow performing impurity profiling of chromatographically unresolved siRNA duplex impurities at the MS level, *e.g.*, as conventionally done for antisense ONs.^14^ However, further studies are recommended. Also, LC-MS analysis revealed that the major species eluting within the chromatographic peak corresponding to siRNA#1 duplex have similar *m*/*z* values (Figure S5a,b), therefore confirming that the shape of the chromatographic peak observed here for siRNA duplexes (*i.e.*, broad and partially resolved into multiple peaks) is indeed derived from partial diastereomer separation. This is an interesting observation that is in line with the recently published finding that ON higher-order structure is the major cause of ON diastereomer separation in high ionic strength-based HILIC.^15^ It is possible that salts are enhancing the stability of siRNAs under IP-RPLC conditions by stabilizing their higher-order structures and that this could be at the base of the increased diastereomer separation observed here. In fact, and illustrated in Figure S6 for two of the compounds employed in the HILIC study,^15^*i.e.*, HS1 (a single-stranded RNA with 4 phosphorothioate linkages and a rigid hairpin structure) and LS3 (a single-stranded RNA with same modifications and base composition as HS1, but with a flexible structure), results observed in HILIC^15^ and salt-mediated IP-RPLC (under same conditions as for Figure 3) are similar. HS1 presents a significant level of diastereomer separation, while such a separation is discreete for LS3.

Despite being desirable in some applications,^16^ diastereomer separation is usually seen as a drawback during ON analysis.^16^ Multiple strategies, including the use of different alkylamines, stationary phases, and eluent cosolvents, have been reported in the literature.^15,16^ As illustrated in Figure 4, such strategies are also applicable to salt-mediated IP-RPLC. For instance, by replacing MeCN with methanol (MeOH)^15^ and using a generic gradient 3, the diastereomer separation illustrated here for siRNA#1 duplex in Figures 2 and 3 is completely suppressed, while the chromatographic resolution between the duplex and excess of antisense strand is maintained. One possible explanation for this observation is that MeOH suppresses hydrogen bonding between the duplex diastereomers and stationary phase.^15^ The same is true for Inclisiran (Figure S7). These outcomes further validate the potential of salt-mediated IP-RPLC for nondenaturing characterization of siRNAs.

**Figure 4:**
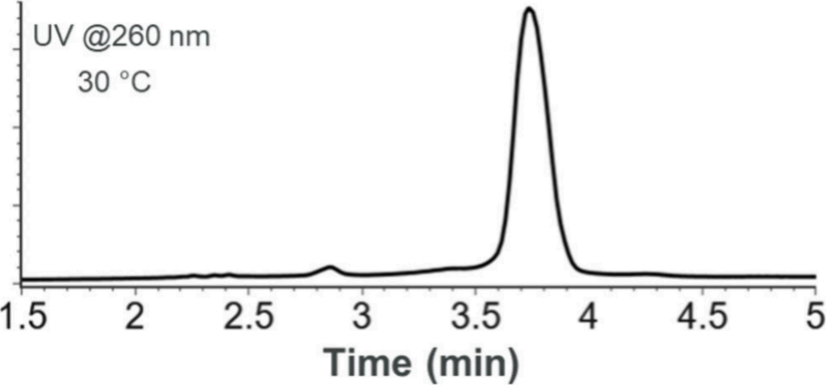
Elution profile at 260 nm of siRNA#1 duplex obtained by replacing MeCN with MeOH in the mobile phases. Column temperature was set to 30 °C.

The study presented here was focused on a limited set of siRNAs and TBuAA-based conditions. Therefore, follow-up studies on a broader range of IPRs, acidic and organic modifiers, salts, column chemistry, and siRNAs of varying design, chemistry, and length are advised. On top of serving the purpose of further validating the efficacy of the strategy described here, these will allow the implementation of a comprehensive model able to predict the impact of any given IP-RPLC condition on the *T*_m_ of any given siRNA, therefore minimizing the number of experiments required during method development.

In conclusion, our results strongly suggest that salts will enable a wider range of IP-RPLC conditions to be evaluated on the merits of improving separation of intact duplex from its impurities, of both double- and single-stranded nature, without the risk of causing duplex denaturation. Therefore, in addition to being simple, our strategy has the potential to (i) transform IP-RPLC into a broadly applicable nondenaturing method for the characterization of siRNAs and (ii) influence future quality control requirements regarding siRNA analytics.

## Experimental Section

Two GalNAc-conjugated siRNA duplexes, named siRNA#1 and Inclisiran (Figure S1), were used. siRNA#1 is an AstraZeneca proprietary sequence with an average mass of 14082.76 Da. HS1 and LS3 described in Lardeux *et al.*(15) were also used here. Stock solutions of siRNA#1 and its single strands were prepared at 100 μM in deionized water. For Inclisiran, duplex and single strands were prepared in 1X PBS. TBuAA and TEtAA were prepared from TBuA (≥99.5%), TEtA (≥99.5), and acetic acid (≥99.8%) purchased from Sigma-Aldrich (St. Louis, MO). AA (LiChropur) was also ordered from Sigma-Aldrich. 1X and 10X Gibco PBS pH 7.4, silicone oil, and LC-MS grade MeCN and MeOH were purchased from Thermo Fisher Scientific (Kandel, Germany). Deionized water (18.2 MΩ cm) was produced using a Milli-Q Integral 3 system (Merck Millipore, Darmstadt, Germany).

For melting temperature measurements, 15.3 μM solutions (90 μL) of siRNA#1 were prepared in representative IP-RPLC diluents (Tables S1 and S2) and transferred to 1 mm path-length cuvettes (ultramicrocell, quartz, stoppered with PTFE stopper). Due to the relatively low boiling point of MeCN, diluent evaporation was expected. To mitigate this, solutions were topped with 10 μL of silicone oil. Cuvettes were thermally equilibrated at 10 °C before starting the measurement cycles from 10 to 90 °C. Experiments were performed on a UV–vis spectrophotometer (Cary 3500 multicell Peltier, Agilent Technologies, Santa Clara, CA). Measurements were taken at 260 nm, with a 2 min hold time, 0.2 °C data intervals, and 0.5 °C min^–1^ heating rate. *T*_m_ values were obtained by calculating the maximum of the first derivative of the melting curves.

For the design of experiments, a mixed-level full-factorial DoE was used to investigate the influence of the MeCN level (16.8 to 50.5 vol %, three levels), the PBS level (0 to 1X, three levels), and the IPR concentration (4.2 and 16.8 mM, two levels) on the *T*_m_ of siRNA#1. The model included three center points. For more details, see Table S2. Multilinear regression was employed for model fitting. Factor coefficients with *p*-value >0.05 were removed to improve the model’s predictive power (*Q*^2^). *Q*^2^ was used as the criterion for model refinement. DoE calculations were conducted using MODDE (Pro version 13, Sartorius Stedim Data Analytics AB, Sweden).

UV-based IP-RPLC analyses of siRNA#1 and Inclisiran were performed on an Agilent Technologies 1100 liquid chromatography system (Agilent Technologies, Santa Clara, CA) equipped with two binary pumps coupled to a diode-array UV detector. An AdvanceBio Oligonucleotide C18, 50 × 2.1 mm, 2.7 μm column (Agilent Technologies) was employed in the analyses. ChemStation was used for data acquisition. Two different sets of 10.6 mM TBuAA mobile phases were prepared for IP-RPLC analysis. For experiments without PBS, mobile phases A (MPA) and B (MPB) were prepared at 25 and 80 vol % MeCN, respectively. Analyses were performed at 8 and 50 °C using a gradient running from 40 to 70%MPB in 6 min followed by a 0.5 min hold and a 4 min re-equilibration step (herein referred to as gradient 1). For experiments using a mobile phase containing 0.25X PBS, MPA was 10 vol % MeCN and MPB 40 vol %. For these conditions, the gradient program ran from 50 to 100%MPB in 6 min followed by a 0.5 min hold and a 4 min re-equilibration step (referred to gradient 2). Analysis was performed at 30, 50, 60, and 65 °C. For all experiments the flow rate was 0.6 mL min^–1^. UV signals were recorded at 260 nm. Each injection consisted of 3 μL of a 5 μM ON solutions.

LC-MS experiments were carried out on an ACQUITY Premier LC system (Waters, Milford, MA) equipped with a binary pump coupled to a photodiode array detector set for UV detection at 260 nm and to a high-resolution mass spectrometer (benchtop RDa TOF, with about 10,000 fwhm mass resolution, Waters). Experiments illustrated in Figures 3, S4, and S6 were performed by using conditions described for gradient 2. For experiments described in Figures 4 and S7, MPA and MPB were prepared at 20 and 100 vol % MeOH, respectively. Analyses were performed using a gradient running from 25 to 65%MPB in 6 min followed by a 0.5 min hold and a 4 min re-equilibration step (herein referred to as gradient 3). All LC-MS experiments were performed using a 0.6 mL min^–1^ flow rate, 30 °C column temperature, and 10.6 mM TBuAA and 39.8 mM AA added to the mobile phases. The mass spectrometer was operated as described in Lardeux *et al.*(15) Each injection consisted of 0.5 μL of 100 μM ON solutions. UNIFI (Waters) was used for the data acquisition and processing.

Except for the LC-MS data, all visualizations were generated using Python and its libraries pandas, numpy, and matplotlib.

## Supporting Information Available

Sequence representation for Inclisiran; experimental *T*_m_ for siRNA#1 under different IP-RPLC diluents, with and without PBS; UV-based chromatograms for Inclisiran at different column temperatures and with salt added to the mobile phases; zoomed-in mass spectra for siRNA#1 duplex, antisense, and sense strands; deconvolved mass spectrum for siRNA#1; UV- and EIC-based chromatograms for siRNA#1; EIC-based chromatograms derived from siRNA single-strands with rigid and flexible structures; UV-based chromatogram for Inclisiran obtained by replacing MeCN with MeOH in the mobile phases (PDF)

## References

[ref1] Hu B.; Zhong L.; Weng Y.; Peng L.; Huang Y.; Zhao Y.; Liang X.-J. Therapeutic siRNA: State of the Art. Signal Transduct. Target. Ther. 2020, 5 (1), 1–25. 10.1038/s41392-020-0207-x. 32561705 PMC7305320

[ref2] Fornstedt T.; Enmark M. Separation of Therapeutic Oligonucleotides Using Ion-Pair Reversed-Phase Chromatography Based on Fundamental Separation Science. J. Chromatogr. Open 2023, 3, 100079 10.1016/j.jcoa.2023.100079.

[ref3] Yogendrarajah P.; Suarez Marina I.; Verluyten W.; Dejaegere E.; Napoletano L.; Boon J.-P.; Hellings M.; Gilar M. Analysis of siRNA with Denaturing and Non-Denaturing Ion-Pair Reversed-Phase Liquid Chromatography Methods. LCGC N. Am. 2023, 41, 60–66. 10.56530/lcgc.na.zs3766l5.

[ref4] Donegan M.; Nguyen J. M.; Gilar M. Effect of Ion-Pairing Reagent Hydrophobicity on Liquid Chromatography and Mass Spectrometry Analysis of Oligonucleotides. J. Chromatogr. A 2022, 1666, 462860 10.1016/j.chroma.2022.462860. 35123169

[ref5] Basiri B.; Murph M. M.; Bartlett M. G. Assessing the Interplay between the Physicochemical Parameters of Ion-Pairing Reagents and the Analyte Sequence on the Electrospray Desorption Process for Oligonucleotides. J. Am. Soc. Mass Spectrom. 2017, 28 (8), 1647–1656. 10.1007/s13361-017-1671-6. 28405940 PMC5569388

[ref6] Beverly M.; Hartsough K.; Machemer L. Liquid Chromatography/Electrospray Mass Spectrometric Analysis of Metabolites from an Inhibitory RNA Duplex. Rapid Commun. Mass Spectrom. 2005, 19 (12), 1675–1682. 10.1002/rcm.1972. 15912467

[ref7] Gilar M.; Redstone S.; Gomes A. Impact of Mobile and Stationary Phases on siRNA Duplex Stability in Liquid Chromatography. J. Chromatogr. A 2024, 1733, 465285 10.1016/j.chroma.2024.465285. 39173502

[ref8] Huang M.; Xu X.; Qiu H.; Li N. Analytical Characterization of DNA and RNA Oligonucleotides by Hydrophilic Interaction Liquid Chromatography-Tandem Mass Spectrometry. J. Chromatogr. A 2021, 1648, 462184 10.1016/j.chroma.2021.462184. 33991753

[ref9] Noll B.; Seiffert S.; Vornlocher H.-P.; Roehl I. Characterization of Small Interfering RNA by Non-Denaturing Ion-Pair Reversed-Phase Liquid Chromatography. J. Chromatogr. A 2011, 1218 (33), 5609–5617. 10.1016/j.chroma.2011.06.057. 21737080

[ref10] Togawa H.; Okubo T.; Nonaka Y.; Yamaguchi T.; Obika S. Retention Behavior of Short Double-Stranded Oligonucleotide and Its Potential Impurities by Anion-Exchange Chromatography under Non-Denaturing Conditions. J. Chromatogr. A 2023, 1691, 463808 10.1016/j.chroma.2023.463808. 36706652

[ref11] Handbook of Analysis of Oligonucleotides and Related Products; Bonilla J. V., Srivatsa S., Eds.; CRC Press: Boca Raton, FL, 2011.

[ref12] McCarthy S. M.; Gilar M.; Gebler J. Reversed-Phase Ion-Pair Liquid Chromatography Analysis and Purification of Small Interfering RNA. Anal. Biochem. 2009, 390 (2), 181–188. 10.1016/j.ab.2009.03.042. 19345196

[ref13] Cecchi T. Ion Pairing Chromatography. Crit. Rev. Anal. Chem. 2008, 38 (3), 161–213. 10.1080/10408340802038882. 28122458

[ref14] Rentel C.; Gaus H.; Bradley K.; Luu N.; Kolkey K.; Mai B.; Madsen M.; Pearce M.; Bock B.; Capaldi D. Assay, Purity, and Impurity Profile of Phosphorothioate Oligonucleotide Therapeutics by Ion Pair–HPLC–MS. Nucleic Acid Ther. 2022, 32 (3), 206–220. 10.1089/nat.2021.0056. 35238617

[ref15] Lardeux H.; Stavenhagen K.; Paris C.; Dueholm R.; Kurek C.; De Maria L.; Gnerlich F.; Leek T.; Czechtizky W.; Guillarme D.; Jora M. Unravelling the Link between Oligonucleotide Structure and Diastereomer Separation in Hydrophilic Interaction Chromatography. Anal. Chem. 2024, 96 (24), 9994–10002. 10.1021/acs.analchem.4c01384. 38855895 PMC11190878

[ref16] Enmark M.; Rova M.; Samuelsson J.; Örnskov E.; Schweikart F.; Fornstedt T. Investigation of Factors Influencing the Separation of Diastereomers of Phosphorothioated Oligonucleotides. Anal. Bioanal. Chem. 2019, 411 (15), 3383–3394. 10.1007/s00216-019-01813-2. 31020370 PMC6543027

