# Supplementary material for: Expanding the Analytical Toolbox for the Nondenaturing Analysis of siRNAs with Salt-Mediated Ion-Pair Reversed-Phase Liquid Chromatography
Source: Anal Chem. 2024 Nov 11;96(47):18590–5. doi: 10.1021/acs.analchem.4c05248 (PMC11603401; doi:10.1021/acs.analchem.4c05248)
Supplement: Supplementary file 1 — ac4c05248_si_001.pdf [file ac4c05248_si_001.pdf]

Supporting Information

Expanding the Analytical Toolbox for the Nondenaturing Analysis of siRNAs with Salt-Mediated Ion-Pair Reversed-Phase Liquid Chromatography

Martin Enmark[1], Ilaria Furlan[2], Porya Habibollahi[2], Christian Manz[3], Torgny Fornstedt[1]*, Jörgen Samuelsson[1]*, Eivor Örnskov[2], Manasses Jora[3]*

[1] Department of Engineering and Chemical Sciences, Karlstad University, Karlstad 651 88, Sweden

[2] Advanced Drug Delivery, Pharmaceutical Sciences, BioPharmaceuticals R&D, AstraZeneca, Mölndal 431 83, Sweden

[3] Medicinal Chemistry, Research and Early Development, Respiratory and Immunology, BioPharmaceuticals R&D, AstraZeneca, Mölndal 431 83, Sweden

*Corresponding authors:
E-mail: Manasses.Jora@astrazeneca.com
E-mail: Jorgen.Samuelsson@kau.se
E-mail: Torgny.Fornstedt@kau.se

TABLE OF CONTENTS

**Figure S1**	Sequence representation and chemical structure of Inclisiran…	S-2
**Table S1**	Experimental data used in the box plot from Figure 1b…	S-3
**Table S2**	Experimental data used in the DoE model from Figure 1c…	S-4
**Figure S2**	Elution profiles of Inclisiran duplex (black), sense strand (blue), and antisense strand (orange)…	S-5
**Figure S3**	Zoomed view between 1500 and 2000 m/z of the mass spectra observed underneath the chromatographic peaks corresponding to (a) siRNA#1 duplex, (b) siRNA#1 antisense strand, and (c) siRNA#1 sense strand…	S-6
**Figure S4**	Deconvolved mass spectrum derived from the mass spectrum acquired for siRNA#1 duplex shown in Figure 3b…	S-7
**Figure S5**	(a) UV-based chromatogram at 260 nm observed for siRNA#1 duplex using a linear gradient between 25 and 40 vol % MeCN in 6 min…	S-8
**Figure S6**	EICs at *m/z* 1661.4 (charge state -4) acquired for single-stranded RNAs (a) HS1 and (b) LS3…	S-9
**Figure S7**	Elution profile at 260 nm of Inclisiran duplex obtained by replacing MeCN with MeOH in the mobile phases…	S-10

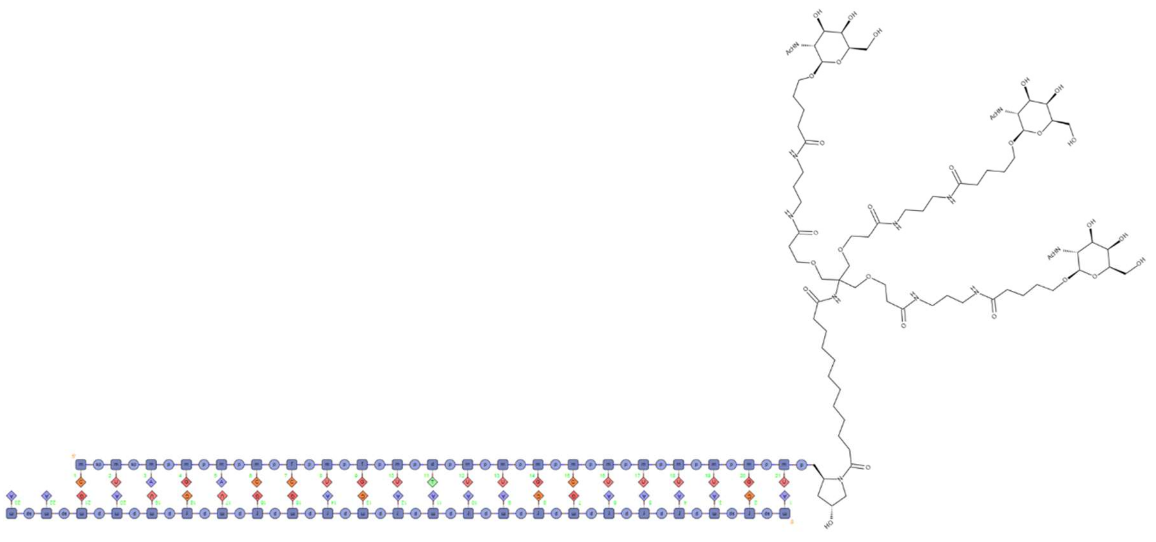

Figure S1. Sequence representation and chemical structure of Inclisiran. m: 2’-O-methyl, f: 2’-fluoro, d: 2’-deoxyribose; p: phosphate, sp: phosphorothioate.

Table S1. Experimental data used in the box plot from Figure 1b. [IPR] corresponds to the concentration of IPR in the diluent. MeCN corresponds to the percent in volume of MeCN in the diluent. Melting temperature corresponds to the temperature derived from the siRNA melting curves.

[IPR] (mM)	MeCN (vol %)	Type of IPR	Melting temperature (°C)
83.0	50.5	TEtAA	46.0
83.0	16.8	TEtAA	45.5
10.6	50.5	TEtAA	37.0
10.6	16.8	TEtAA	31.0
16.8	50.5	TBuAA	26.2
16.8	16.8	TBuAA	24.2
4.2	50.5	TBuAA	29.2
4.2	16.8	TBuAA	13.6

Table S2. Experimental data used in the DoE model from Figure 1c. [IPR] corresponds to the concentration of TBuAA in the diluent. MeCN corresponds to the percent in volume of MeCN in the diluent. PBS corresponds to 1X phosphate-buffered saline, with 0 meaning no PBS added, 1 meaning 1X PBS added, and 0.5 meaning two-times diluted 1X PBS added to the diluent. Melting temperature corresponds to the temperature derived from the siRNA melting curves. *indicates center points of the DoE model. **corresponds to additional experiments performed to enable three-level modeling of PBS and MeCN.

[IPR] (mM)	MeCN (vol %)	PBS (X)	Melting temperature (ºC)
16.8	50.5	1	84.6
16.8	50.5	0	26.2
16.8	16.8	1	71.6
4.2	50.5	1	85.0
16.8	16.8	0	24.2
4.2	16.8	1	71.8
4.2	50.5	0	29.2
4.2	16.8	0	13.6
10.6	33.7	0.5	70.2*
10.6	33.7	0.5	70.4*
10.6	33.7	0.5	70.6*
10.6	50.5	0.5	84.0**
10.6	16.8	0.5	65.2**
10.6	33.6	1	76.0**
10.6	33.6	0	16.2**

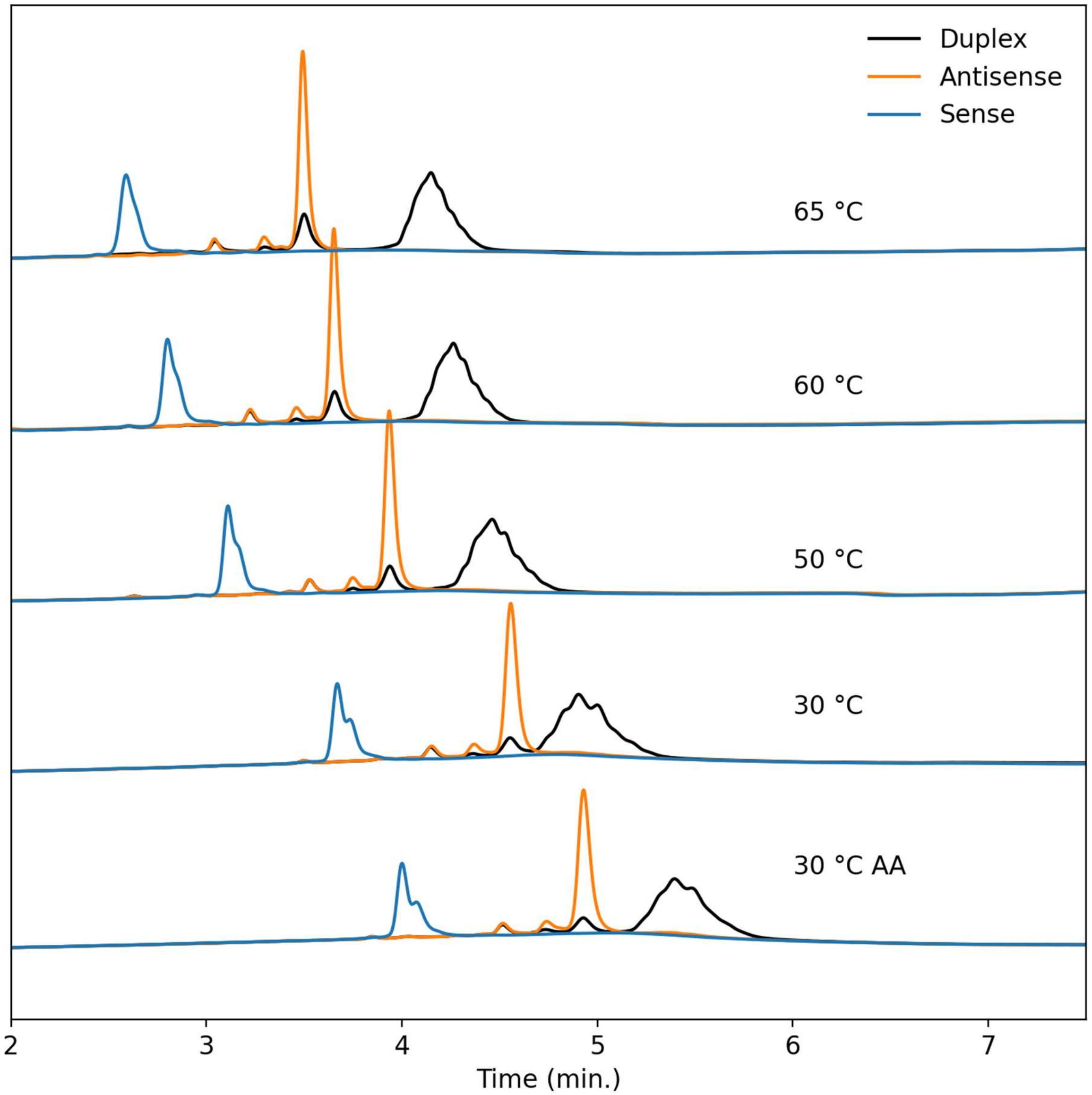

Figure S2. Elution profiles of Inclisiran duplex (black), sense strand (blue), and antisense strand (orange) obtained with 0.25X PBS in the mobile phases, gradient 2, 0.6 mL min^{-1} flow rate, and at column temperatures of 30 °C, 50 °C, 60 °C, and 65 °C. The bottom trace at 30 °C was acquired with 39.8 mM AA in the mobile phase instead of PBS.

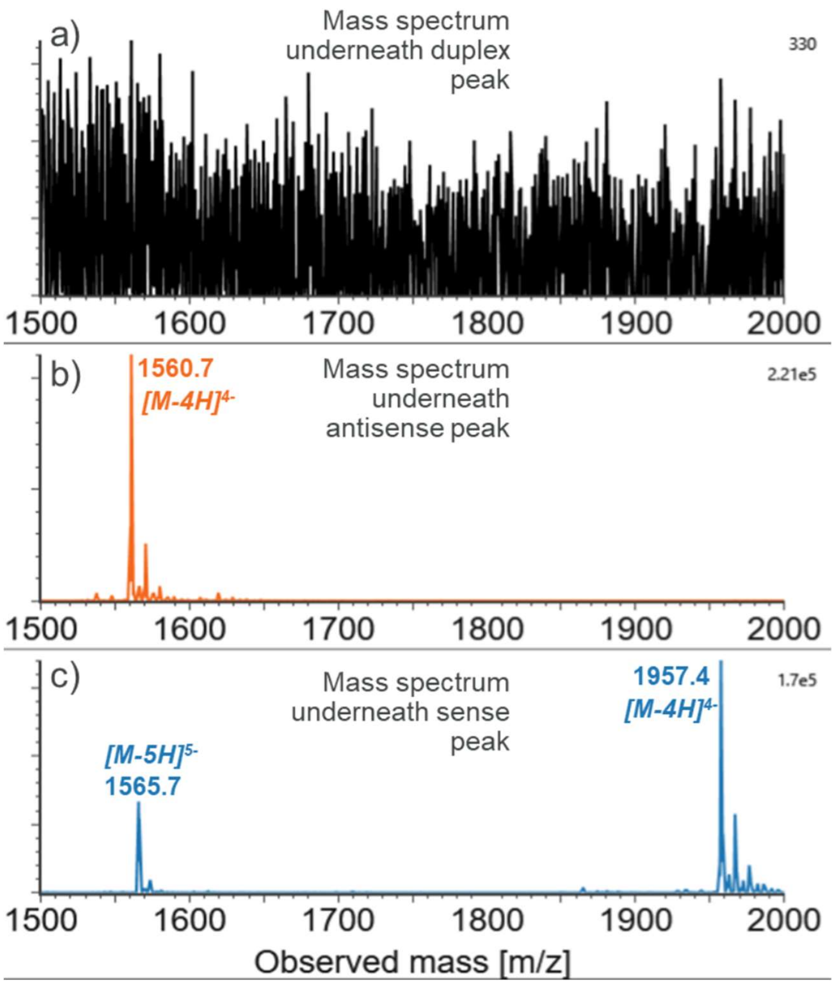

Figure S3. Zoomed view between 1500 and 2000 *m/z* of the mass spectra observed underneath the chromatographic peaks corresponding to (a) siRNA#1 duplex, (b) siRNA#1 antisense strand, and (c) siRNA#1 sense strand. The intensity of each mass spectrum was normalized according to the most abundant peak within each individual mass spectrum and within the selected *m/z* range.

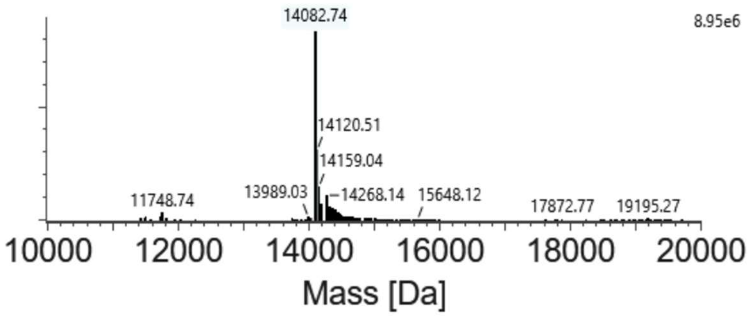

Figure S4. Deconvolved mass spectrum derived from the mass spectrum acquired for siRNA#1 duplex shown in Figure 3b. Deconvolution was performed in UNIFI using BayesSpray algorithm. The deconvolution parameters included: input *m/z* range of 2000-4000, output average mass option checked, charge state range of 4-7, resolution of 10000, molecule type set to Oligonucleotide, and iterations and objects set to 50 and 3, respectively. All other parameters remained at their default settings.

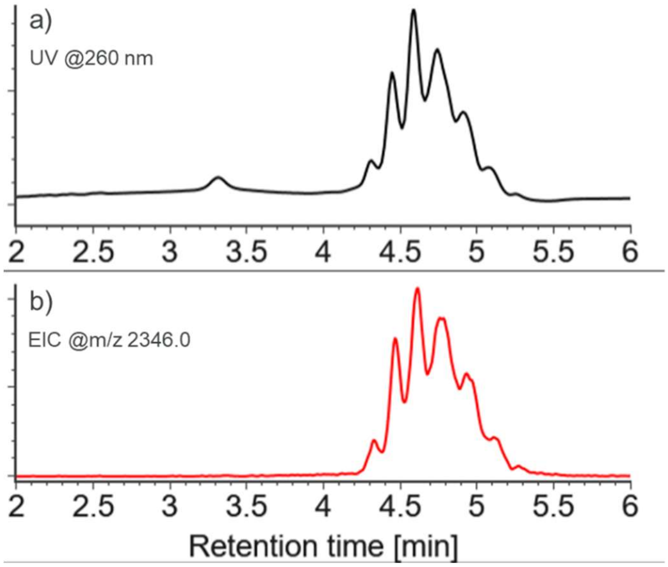

Figure S5. (a) UV-based chromatogram at 260 nm observed for siRNA#1 duplex acquired using gradient 2. Flow rate was set to 0.6 mL min^{-1} and column temperature was set to 30 °C. (b) EIC at *m/z* 2346.0, which corresponds to siRNA#1 at its most abundant charge state, *i.e.*, -4.

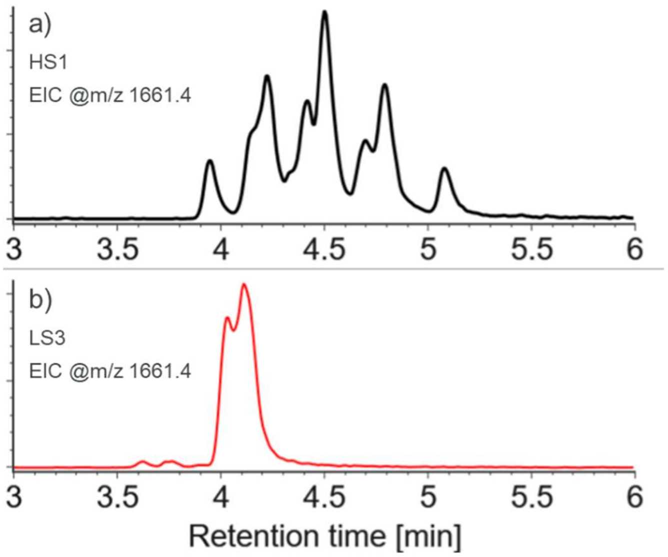

Figure S6. EICs at *m/z* 1661.4 (charge state -4) acquired for single-stranded siRNAs (a) HS1 and (b) LS3 using gradient 2. Flow rate was set to 0.6 mL min^{-1} and column temperature was set to 30 °C. HS1 is a representative modified single-stranded siRNA with 4 phosphorothioate linkages and a rigid hairpin structure, and LS3 is a single-stranded siRNA with same modifications and base composition as HS1, but with a flexible structure.

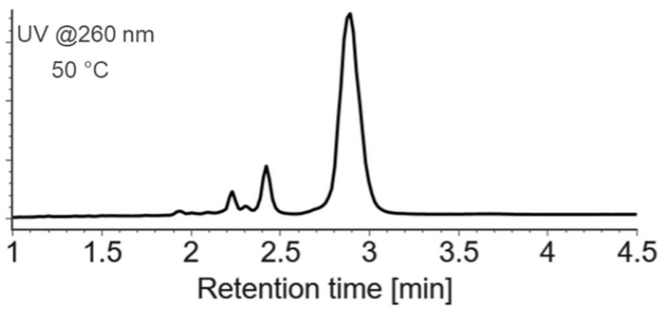

Figure S7. Elution profile at 260 nm of Inclisiran duplex obtained by replacing MeCN with MeOH in the mobile phases. Column temperature was set to 50 °C.
